# Generalized epilepsy in a patient with mosaic Turner syndrome: a case report

**DOI:** 10.1186/1752-1947-8-109

**Published:** 2014-04-02

**Authors:** Kai-Ming Jhang, Tung-Ming Chang, Ming Chen, Chin-San Liu

**Affiliations:** 1 Department of Neurology, Changhua Christian Hospital, 135 Nanhsiao Street, 500 Changhua, Taiwan; 2 Vascular and Genomic Center, Changhua Christian Hospital, 135 Nanhsiao Street, 500 Changhua, Taiwan; 3 Graduate Institute of Integrated Medicine, College of Chinese Medicine, China Medical University, Taichung 404, Taiwan; 4 Department of Pediatric Neurology, Changhua Christian Hospital, Changhua, Taiwan; 5 Department of Genomic Medicine, and Center for Medical Genetics, Changhua Christian Hospital, Changhua, Taiwan; 6 Department of Obstetrics and Gynecology, National Taiwan University, Taipei, Taiwan; 7 Department of Life Sciences, National Chung-Hsing University, Taichung, Taiwan; 8 Department of Pediatrics, and Department of Medical Genetics, College of Medicine and Hospital, National Taiwan University, Taipei, Taiwan; 9 Department of Life Sciences, Tunghai University, Taichung, Taiwan; 10 College of Medicine, Chung-Shan Medical University, Taichung, Taiwan

**Keywords:** Asymmetrical lateral ventricles, Epilepsy, Mosaic, Turner syndrome

## Abstract

**Introduction:**

Reports on cases of epilepsy in Turner syndrome are rare and most of them have cortical developmental malformations. We report the case of a Taiwanese patient with mosaic Turner syndrome with generalized tonic–clonic epilepsy and asymmetrical lateral ventricles but no apparent cortical anomaly.

**Case presentation:**

A 49-year-old Taiwanese woman without family history presented with infrequent generalized tonic–clonic epilepsy since she was 11 years old. On examination, her short stature, webbed neck, swelling of hands and feet, retrognathic face, and mild intellectual disability were noted. She had spontaneous menarche and regular menses. Brain magnetic resonance imaging showed asymmetrical lateral ventricles and diffuse subcortical white matter T2-weighted hyperintensities. Chromosome studies disclosed low aneuploid (10%) 45,X/46,XX/47,XXX mosaic Turner syndrome.

**Conclusions:**

There is increasing evidence that epilepsy can be an uncommon presentation of Turner syndrome. Mosaic Turner syndrome with 47, XXX probably increases the risk of epilepsy but more research is needed to reach a conclusion. This case also strengthens our knowledge that Turner syndrome can be one of the pathologic bases of asymmetrical lateral ventricles. When a patient has idiopathic/cryptogenic epilepsy or asymmetrical lateral ventricles on brain images, the presence of a mild Turner phenotype warrants further chromosome studies.

## Introduction

Turner syndrome (TS) results from the absence of one X chromosome. About 45% of postnatal patients with TS are apparently nonmosaic monosomy X, whereas the remaining have structural chromosome abnormality or mosaicism [1]. The classic phenotype includes short stature, webbing of the neck, gonadal dysgenesis, vascular abnormalities and variable somatic stigmata. Patients with TS may have a spectrum of neuropsychiatric problems, including autism, attention-deficit hyperactivity disorder, schizophrenia, and cognitive difficulties especially visual–spatial, mathematical, and social skills [2]. There are only a few case reports of patients with TS who have epilepsy, and they are frequently associated with malformations of cortical development [3-12]. We report the case of a Taiwanese patient with mosaic TS and generalized tonic–clonic epilepsy and asymmetrical lateral ventricles (ALV).

## Case presentation

A 49-year-old Taiwanese woman presented with infrequent generalized seizures since childhood. She had her first generalized tonic–clonic seizure attack when she was 11 years old. The duration was 1 to 3 minutes with short postictal state. The frequency was about one to two attacks every decade under carbamazepine 200mg twice daily and valproic acid 500mg twice daily. She stopped both antiepileptic drugs against medical advice 4 years ago and started taking a Chinese herb. However, she presented with another episode of generalized tonic–clonic seizure and was transferred to our neurology out-patient department.

**Figure 1:**
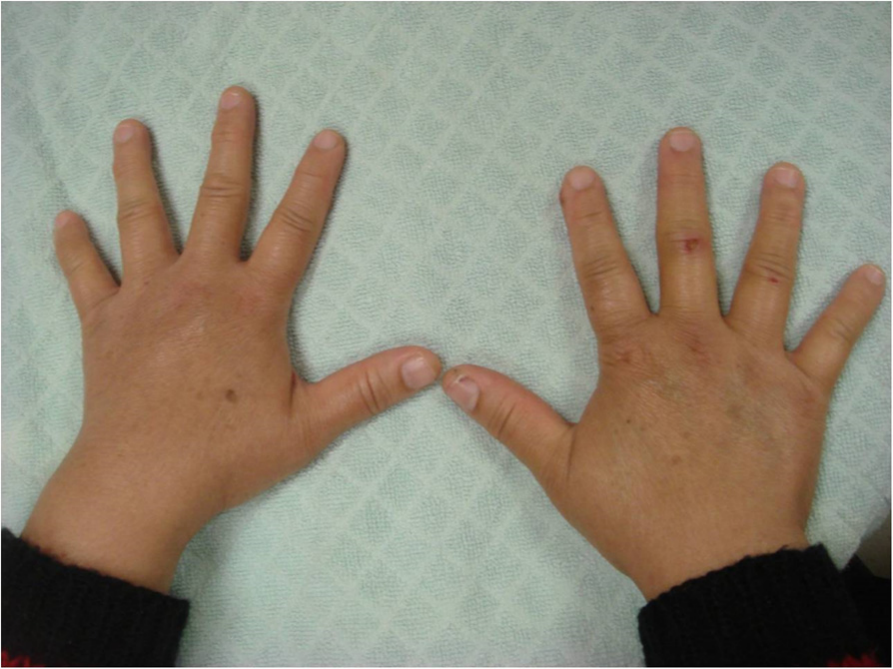
**Hands of the patient.** Swelling of both hands and small fingernails.

**Figure 2:**
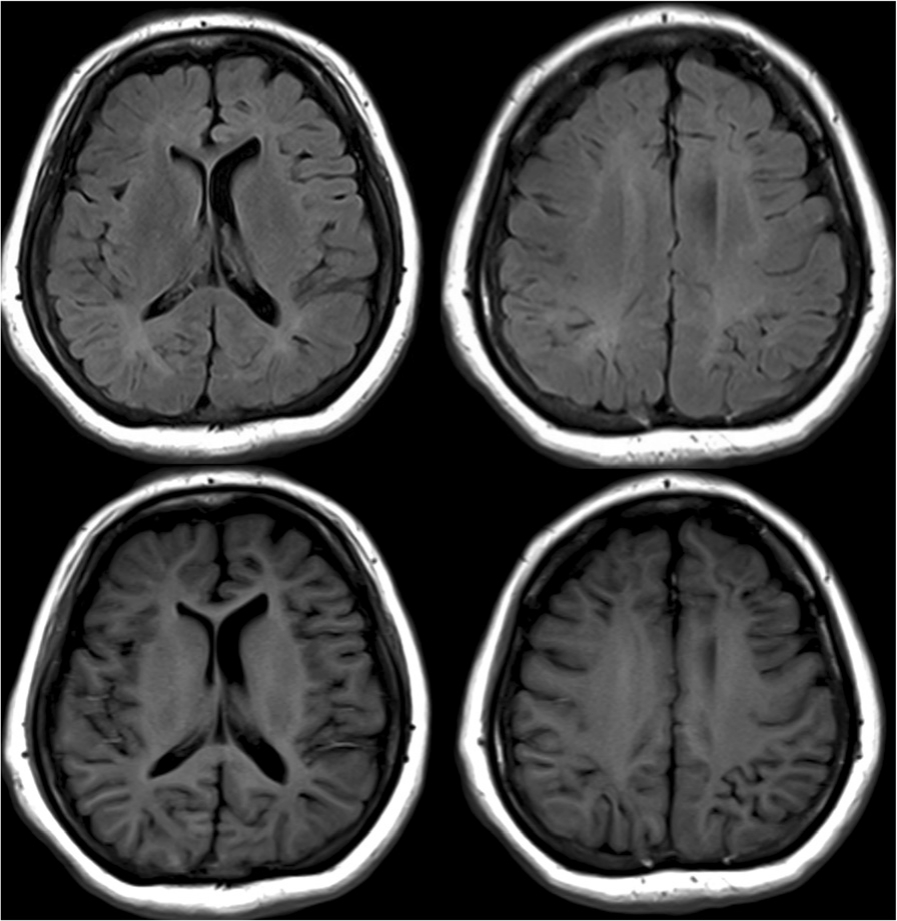
**Brain magnetic resonance imaging.** Axial T2-weighted fluid-attenuated inversion recovery image (above) and T1-weighted image (below) at different level showed prominent smaller right lateral ventricles. Diffuse periventricular and subcortical white matter hyperintensities are also noted.

**Figure 3:**
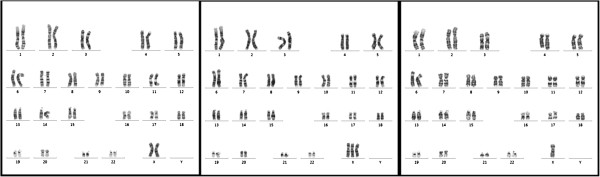
**G-band chromosomal analysis of 30 peripheral blood lymphocytes revealed 45,X[2]/47,XXX[1]/46,XX[27] mosaic turner genotype.**

She had been born smoothly without abnormal birth history. Her family history of epilepsy or hereditary diseases is unremarkable. She has mild intellectual disability and completed basic national education only (junior high school). She had spontaneous pubarche, thelarche, and menarche at 13 years old. Her menstrual cycles were about 28 days and generally regular and she began menopause at 45 years old. She did not get married and had no sexual experience. She has a medical history of hyperlipidemia, thyroid adenomatous goiter, and gastric polyp. A physical examination showed her height 1.46m (1st percentile compared to same age population in Taiwan; height of her mother was 1.53m), weight 64kg, swelling of hands and feet, small fingernails (Figure 1), retrognathic face, webbed neck, and secondary sexual characteristics Tanner V. Her neurological examination was essentially normal. Laboratory data revealed normal thyroid functions. Brain magnetic resonance imaging (MRI) revealed asymmetrical bilateral ventricular size (right side smaller), and bilateral periventricular and subcortical white matter hyperintensity (Figure 2). A pelvic ultrasound showed anteverted uterus of 4.8 × 5.2 × 4.2cm, right adnexal cyst of 3.1 × 4.3 × 4.2cm, and invisible left ovary. An electroencephalograph (EEG) showed 10 to 20uV beta rhythm with reactive 20 to 30uV posterior 10 to 11Hz alpha background activities without recording of epileptiform discharges. A G-band chromosomal analysis on peripheral blood lymphocytes reported 45,X[2]/47,XXX[1]/46,XX[27] (Figure 3), consistent with mosaicism TS. Cardiac echo showed mild mitral and tricuspid regurgitations without abnormal aortic valve. An X-ray of her hand showed normal alignment without shortening of the fourth metacarpal bone.

## Discussion

We reported the case of a middle-aged woman with mosaic 45X/46,XX/47,XXX TS and generalized tonic–clonic epilepsy. Reports on epilepsy in TS are rare [3-12]. Symptomatic epilepsy with cortical developmental malformations, including pachygyria and lissencephaly [9], bilateral frontal polymicrogyria [5], bilateral perisylvian hypoplasia [4], has been reported in patients with TS. Other cases of TS with epilepsy had no evidence of structure abnormality [3,7,12]. The chromosome anomalies included 45,X[4], 45,X/46,XX [5,7], 45,X/47,XXX [9], deletion of short-stature homeobox gene [3], deletion of the whole short arm of the X chromosome [10], partial monosomy Xq (Xq23 → qter) [12], and proposita (45,X/46,XXp+/47,XXp + Xp +) [8]. Grosso *et al*. reviewed seizures and EEGs in 43 patients with polyploidy/aneuploidy of the X chromosome of which 11 patients with TS (two with mosaic 45,X/46,XX) did not have epilepsy [11]. However, patients with triple X syndrome or gross X-autosomal rearrangement were associated with epilepsy. Most of the patients presented with complex partial seizures. Similar findings have been reported by other groups. Approximately 15% of patients with trisomy X syndrome have seizure disorders [13]. Types of seizure range from absence, partial to generalized seizures, with good responses to standard anticonvulsant treatments. Our patient has both monosomic (6.7%) and trisomic (3.3%) X chromosomes in peripheral blood cells. Although the percentage is low, the component of trisomy X probably increases the incidence of seizure disorder and contributes partly to her generalized tonic–clonic epilepsy. There are only case reports discussing seizures in 45,X/46,XX/47,XXX mosaic TS, probably because this karyotype comprises only 3% of total patients with TS [14]. More research is needed to confirm if the prevalence of epilepsy is higher in patients with TS and triple X cell line.

The pathophysiology of epilepsy in TS is unclear. The X chromosome plays an important role in cerebral development. Significant differences of gray matter volumes and alterations of neuronal network are observed in regional areas of the brain including parietal, prefrontal cortex, and amygdalo-hippocampal structures [2,15]. These changes are associated with cognitive dysfunction and possibly explain partly the mechanism of epilepsy. About 10% of patients with TS have clinical intellectual disability, but most patients with TS experience difficulties of higher-order visual–spatial functions, arithmetical abilities, executive function, and specific aspects of language [2,14]. In patients with mosaic 45,X/46,XX/47,XXX, the proportion of intellectual disability ranges from 0 to 13%, which was considered to be similar to monosomy X [14,16]. Previous literature found that in trisomy X syndrome, patients with epilepsy have a higher rate of intellectual disability than patients without epilepsy [11,17]. The present case has clinically evident intellectual disability. The relationship between epilepsy and trisomy X chromosome may probably correlate to her intellectual impairment.

Patients with triple X mosaicism have a phenotype that differs from the classic 45,X TS, more (about one-quarter) were diagnosed in adult life, they have an absence of edema in infancy, a higher percentage of spontaneous menarche (60% verses 10%), a lower rate of sexual infantilism (90% verses 50%), and lower cardiac and renal malformations [14,16,18]. The uniform feature in TS with or without mosaicism is short stature. About 21% of patients with mosaicism have consistent menses in adulthood [18]. This case had normal spontaneous menarche and regular menses until 45 years old; however, her reproductive capability is uncertain due to her unmarried status. Another factor probably related to her mild phenotype is only 10% of aneuploid cells. Previous literature indicates that more than 6% of aneuploidy is responsible for clinical changes, with body mass index positively and menarche age inversely correlated with the percentage of 45,X cells [19]. The mean age of diagnosis of ovarian failure is significantly older in patients with low-level X chromosome mosaicism (6 to 10% of aneuploid cells) than in those with higher aneuploidy [20]. The low aneuploidy in the present patient probably contributed to her mild phenotype and persistent regular menstruation.

Another striking feature in the present case is lateral ventricular asymmetry. To the best of our knowledge, no previous report has correlated both conditions. ALV are not uncommon (it was found in 5 to 12% patients received head computed tomography due to various indications) and are associated with headache, seizures and positive human immunodeficiency virus status [21,22]. One case with ALV has been found to have trisomy 21; however, no obvious pathology was found in most patients [23]. ALV was considered not pathological but probably a normal variation [23,24]. We report the first case of ALV with underlying TS pathology.

White matter hyperintensities have only rarely been reported in patients with TS. Only one case report, a 52-year-old 45,X/46,XX Japanese woman, demonstrated the same findings as our patient [25]. Diffusion tensor imaging reveals that the microstructures of the white matter of young patients with TS and normal controls differ [26]. Alterations in the white matter pathway correlated with the cognitive dysfunction. Both our case and the reported one are middle-aged women, and it is not surprising that normal age-related white matter change can occur. Another explanation could be ischemic lesions because she has vascular risk factors including hyperlipidemia and obesity. The relationship between TS and MRI white matter lesions is still vague.

## Conclusions

We reported the case of a 49-year-old woman with low aneuploid 45,X/46,XX/47,XXX mosaic TS, having short stature, generalized epilepsy, ALV, and no obvious premature ovarian failure or cortical malformations. There is increasing evidence that epilepsy can be an uncommon presentation of TS. Mosaic TS with 47,XXX probably increases the risk of epilepsy but more research is needed to reach a conclusion. This case also strengthens our knowledge that TS can be one of the pathologic bases of ALV. When a patient has idiopathic/cryptogenic epilepsy or ALV on brain images, the presence of a mild Turner phenotype warrants further chromosome studies.

## Consent

Written informed consent was obtained from the patient for publication of this case report and any accompanying images. A copy of the written consent is available for review by the Editor-in-Chief of this journal.

## Abbreviations

ALV: Asymmetrical lateral ventricles; EEG: Electroencephalograph; MRI: Magnetic resonance imaging; TS: Turner syndrome.

## Competing interests

The authors declare that they have no competing interests.

## Authors’ contributions

CSL analyzed and interpreted the patient data; KMJ was a major contributor in writing the manuscript. TMC provided idea of discussion MC provided the analysis of the genetic report. All authors read and approved the final manuscript.
